# Magnetic Resonance Imaging Safety Knowledge, Attitudes and Practices Among Radiology Nurses in Saudi Arabia: A National Multicentre Study

**DOI:** 10.1002/nop2.70677

**Published:** 2026-07-15

**Authors:** Mohammed A. Alolayani, Dhafer M. Alahmari, Wejdan M. Arif, Othman I. Alomair, Sami A. Alghamdi

**Affiliations:** ^1^ Radiological Sciences DepartmentCollege of Applied Medical Sciences King Saud University Riyadh Saudi Arabia; ^2^ Medical Imaging Department, Research Centre King Saud Medical City Riyadh Saudi Arabia

**Keywords:** KAP, MRI safety, nurses, radiology department, Saudi Arabia

## Abstract

**Aim:**

To evaluate magnetic resonance imaging (MRI) safety‐related knowledge, attitudes and practices (KAP) among nurses working in radiology departments across Saudi Arabia and to identify independent predictors of MRI safety knowledge.

**Design:**

A national multicentre cross‐sectional study.

**Methods:**

A national multicentre cross‐sectional study was conducted among 153 radiology nurses using a structured self‐administered questionnaire to assess socio‐demographic characteristics and MRI safety‐related KAP. Descriptive statistics summarized the response distributions. Inferential analyses included chi‐square tests, *t*‐tests, ANOVA and multiple linear regression to determine the predictors of knowledge scores.

**Results:**

High correct response rates were observed for operational safety items, including metallic object screening (95.4%), projectile hazards (87.6%), MRI‐compatible equipment use (90.2%) and hearing protection requirements (85.6%) (all *p* < 0.0001). Conceptual gaps were identified: only 54.9% correctly recognized that MRI does not use ionizing radiation, and 51.6% correctly understood that pregnant staff are not automatically prohibited from working in MRI departments. Attitude and practice responses showed strong agreement across items (*p* < 0.0001). Knowledge scores differed significantly by age group (*p* = 0.017), hospital sector (*p* = 0.039), daily MRI patient contact (*p* < 0.001) and formal MRI safety training (*p* < 0.001). In multivariable analysis, older age, daily MRI patient contact and formal MRI safety training remained independent predictors of knowledge scores, with the model explaining 21.6% of the variance (*R* ^2^ = 0.216).

**Conclusion:**

Radiology nurses demonstrated strong self‐reported MRI safety practices and positive safety attitudes; however, selected conceptual knowledge gaps remain. Structured and periodic MRI safety education may strengthen radiology nursing competency and support institutional MRI safety performance.

**Patient or Public Contribution:**

No patient or public contribution.

## 1 Introduction

Magnetic resonance imaging (MRI) is an indispensable diagnostic modality that produces high‐resolution anatomical images without exposing patients to ionizing radiation (Hartwig et al. 2009). It operates through the interaction of strong static magnetic fields, rapidly switching gradient fields and radiofrequency (RF) pulses, yielding superior soft‐tissue contrast and diagnostic accuracy (Carr and Grey 2002; Karunarathna et al. 2025; Sule and Ampofo 2025). Despite its clinical value, the MRI environment presents distinctive safety challenges, including the projectile effect on ferromagnetic materials, RF‐induced tissue heating and acoustic noise generated by gradient coil switching. These hazards demand rigorous adherence to established safety protocols and the development of a robust culture of safety within radiology departments (Alghamdi et al. 2023; Smith 2010; Stafford 2020; Stogiannos and Westbrook 2020). Failure to comply with established MRI safety standards has been shown to be associated with preventable adverse events, including projectile incidents and RF‐related thermal injuries, highlighting the importance of continuous safety education and structured oversight (Dempsey et al. 2001; Shah and Aran 2023).

Within this framework, nurses play a pivotal role in maintaining MRI safety. They are responsible for screening patients for contraindications, ensuring the removal of metallic objects, verifying the MRI conditionality of implants and external devices, monitoring patients during scanning and assisting in emergencies. These responsibilities are emphasized in the American College of Radiology (ACR) Manual on MR Safety, which highlights that nurses form a critical component of the MRI safety chain through their performance of rigorous pre‐scan screening, continuous visual and verbal monitoring, and prompt responses to adverse events (ACR Committee on MR Safety et al. 2020). Evidence also indicates that effective screening, patient education and vigilant monitoring by nursing staff are associated with improved MRI safety practices and reduced risk of preventable adverse events (Abd Aziz et al. 2022). International research further demonstrates that healthcare professionals who receive structured MRI safety training exhibit significantly higher preparedness and confidence in maintaining safe MRI environments (Mittendorff et al. 2023).

Saudi Arabia is no exception regarding the need for robust and comprehensive MRI safety practices. Various nationwide studies have provided insight into these practices in the country. For example, Alghamdi (2023) surveyed 113 hospitals and found that although most MRI units comply with ACR guidelines, over half lacked a magnetic resonance safety officer (MRSO) and regular safety training. In a subsequent study, Alghamdi (2024) identified widespread misconceptions among patients regarding MRI safety, including confusion about radiation and pregnancy, and reported that healthcare providers, including nurses in MRI settings, were the most common source from which they obtained MRI‐related information. These findings underscore both institutional commitments to ensuring MRI safety and persistent educational gaps at the staff–patient interface. Kanbayti (2025) observed limited MRI safety awareness among physicians and nurses in Jeddah, Saudi Arabia, particularly concerning projectile hazards and contrast safety; however, that study was limited to a single city and did not include multiple centres across the country. Another study conducted in the Saudi Arabian city of Tabuk among 105 nurses reported that only 26.7% demonstrated high knowledge of MRI safety standards, whereas 77.1% showed strong adherence to safety protocols. This apparent discrepancy may reflect the distinction between procedural compliance and conceptual knowledge, where nurses follow established safety protocols and institutional guidelines without necessarily possessing comprehensive theoretical understanding of MRI safety principles. The authors concluded that limited knowledge of MRI safety standards may increase the likelihood of adverse incidents and emphasized the need for continuous education and institutional training programmes (Alghamdi et al. 2021).

International research reveals similar patterns. For example, in Jordan, Al‐Radaideh and Al‐Modallal (2023) found variable knowledge of MRI safety among nurses and MRI technologists, with formal training being a predictor of better knowledge. Meanwhile, in Malaysia, Abd Aziz et al. (2022) reported that fewer than one‐quarter of healthcare workers demonstrated good knowledge of MRI safety. Comparable findings have also been reported in Australia and New Zealand, where variability in MRI safety preparedness among *radiographers* was linked to differences in exposure to formal training and continuing professional development (Mittendorff et al. 2023).

Despite the existence of clear national and international guidelines, MRI safety performance depends largely on the knowledge and practices of healthcare professionals directly involved in MRI procedures, particularly nurses. In Saudi Arabia, only fragmented data on this topic have been collected, limited to single institutions, specific geographic regions, patient populations or mixed healthcare professional groups. To the best of our knowledge, no study has comprehensively evaluated nurses' knowledge, attitudes and practices (KAP) regarding MRI safety across the country. To address this gap, the present study was conducted as the first multiregion, multisector (public vs. private), multicentre national assessment of MRI safety‐related KAP among nurses in Saudi Arabia. Specifically, the study aimed to evaluate MRI safety‐related KAP among nurses working in radiology departments across Saudi Arabia and to identify independent predictors of MRI safety knowledge. It was hypothesized that MRI safety‐related knowledge, attitudes and practices would vary according to participants' demographic and professional characteristics.

## 2 Methods

### 2.1 Study Design

This cross‐sectional study was conducted between December 2025 and January 2026 across 15 hospitals in Saudi Arabia to evaluate nurses' KAP concerning MRI safety. The study design and questionnaire were informed by previously published national and regional MRI safety surveys (Abd Aziz et al. 2022; ACR Committee on MR Safety et al. 2020; Al‐Radaideh and Al‐Modallal 2023; Alghamdi 2023). The questionnaire was adapted from these surveys and modified to align with the objectives of the present study. Specifically, the instrument was tailored to nurses working in radiology departments; items not directly applicable to this population were excluded, and additional items addressing MRI safety training, frequency of MRI patient contact and MRI‐related professional responsibilities were incorporated. Prior to distribution, the questionnaire was reviewed by an academic with expertise in biostatistics and survey‐based research to ensure the clarity, relevance and appropriateness of the questionnaire items. Hospitals were selected to represent the major healthcare sectors in the country and to ensure broad geographical coverage across the central, eastern, western, northern and southern regions of Saudi Arabia. The sample included 10 public hospitals (2 university hospitals, 1 military hospital, 1 National Guard hospital and 6 Ministry of Health hospitals) and five private hospitals.

The questionnaire comprised four sections. The first captured socio‐demographic and professional variables (age, sex, education, years of experience, sector [public or private], region and prior MRI safety training). The second assessed knowledge of core MRI safety principles, including static‐field hazards and projectile risks, gradient‐related acoustic noise, RF heating and burns, device and implant compatibility/conditionality, pregnancy‐related precautions and emergency preparedness. The third evaluated attitudes toward MRI safety culture, institutional support, interprofessional communication and willingness to engage in training. The fourth examined self‐reported practices within MRI environments, focusing on screening adherence, zone/access control, patient protection and monitoring, and incident preparedness. Items in the third and fourth sections were rated using a five‐point Likert scale ranging from ‘strongly disagree’ to ‘strongly agree’.

### 2.2 Sample Size and Eligibility

Given that there are 2–15 nurses working in radiology in the 15 hospitals included in this study, the accessible population was estimated at 30–225 nurses. The sample size was calculated using the standard single‐proportion formula: *n* = *Z* ^2^ *p*(1 − *p*)/*d* ^2^, where *Z* represents the standard normal deviate corresponding to the desired confidence level, *p* is the estimated proportion, and *d* is the margin of error. Using a 95% confidence level (*Z* = 1.96), 5% margin of error (*d* = 0.05) and an assumed proportion of 0.50 (*p* = 0.50), the initial required sample size was 385. After applying finite population correction using an estimated midpoint population (*N* ≈ 128), the minimum required sample was 96. Allowing for a 20% non‐response rate, the target sample was set at 120.

Inclusion criteria comprised working as a registered nurse in a radiology department and having responsibilities involving MRI patient care or safety‐related activities, either routinely or as part of rotational duties, and providing informed consent to participate in this study. Exclusion criteria included incomplete responses and working as a trainee, nursing intern, unlicensed staff member or nurse not assigned to a radiology department. A total of 153 nurses were included in the final analysis, exceeding the calculated minimum requirement.

### 2.3 Data Collection

The survey was administered electronically using Google Forms. To achieve the recruitment target, a description of the study, a call for participation and a link to the survey were disseminated via hospital e‐mail systems, nursing supervisors and professional communication platforms to nurses working in radiology departments across the participating hospitals. A consecutive sampling strategy was not used; instead, all eligible nurses working in radiology departments at the participating hospitals were invited to participate voluntarily. Participation was voluntary and anonymous, and electronic informed consent was obtained prior to accessing the questionnaire. No personal identifiers were collected. After the initial distribution of the survey link, a single reminder was sent to the nurses 2 weeks later to enhance the response rate.

Among individuals who accessed the survey, three declined participation by selecting ‘No’ on the electronic consent page, while additional responses were not included in the final analysis because of incomplete questionnaire completion. Because the survey link was distributed through multiple communication channels, the exact number of eligible nurses who received the invitation could not be verified; therefore, the recruitment denominator and response rate could not be determined.

### 2.4 Ethical Considerations

Ethical approval was obtained from the Institutional Review Board (IRB) of King Saud University Medical City, King Saud University, Riyadh, Saudi Arabia (IRB No. E‐25‐10522; approval granted on 07 January 2026). The study adhered to the principles of the Declaration of Helsinki. Participation was completely voluntary, and all responses remained confidential and were used solely for research purposes.

### 2.5 Statistical Analysis

Data were coded and analysed using the Statistical Package for the Social Sciences (SPSS), version 26.0 (IBM Corp., Armonk, NY, USA). Descriptive statistics (frequencies, percentages, mean and standard deviation) were used to summarize socio‐demographic and professional characteristics.

Responses to items relating to MRI safety‐related KAP are presented as frequencies and percentages. For the knowledge domain, correct responses were assigned a score of 1, whereas incorrect and ‘I do not know’ responses were assigned a score of 0. Composite KAP scores were calculated by summing individual item responses. Internal consistency reliability was assessed using Cronbach's alpha for the knowledge, attitude and practice domains.

Prior to the inferential analyses, the assumptions for parametric testing were evaluated. Normality of composite KAP scores was assessed using the Shapiro–Wilk test and visual inspection of histograms and Q–Q plots. Homogeneity of variances was examined using Levene's test. As no substantial violations were identified, parametric tests were considered appropriate.

The chi‐square test for homogeneity was used to examine differences in the distribution of categorical responses across KAP items. Differences in mean composite scores across socio‐demographic and professional characteristics were analysed using independent samples *t*‐tests for two‐group comparisons and one‐way analysis of variance (ANOVA) for variables with more than two categories. Effect sizes were calculated using Cohen's *d* for *t*‐tests and eta squared (*η* ^2^) for ANOVA to determine the magnitude of group differences. When ANOVA results were statistically significant, Tukey's honestly significant difference post hoc tests were performed to identify specific between‐group differences.

Multiple linear regression analysis was conducted to identify factors independently associated with composite knowledge scores. Categorical predictors were entered into the regression model using dummy coding. Statistical significance was set at *p* < 0.05 for all analyses.

## 3 Results

### 3.1 Socio‐Demographic and Professional Characteristics of Nurses

A total of 158 nurses responded to the survey. After excluding five responses that were incomplete or for which consent was not granted, 153 questionnaires were included in the final analysis (completion rate: 96.8%). More than half of the participants (57.5%) were aged 30–39 years. The majority of respondents (66.7%) were female.

Regarding educational qualifications, most nurses (71.2%) held a bachelor's degree. A large proportion of nurses (80.4%) were employed in public hospitals. Most participants were based in the central region of Saudi Arabia (59.5%).

In terms of professional experience, 35.9% of nurses had worked in radiology for more than 6 years. Over half of the nurses (52.9%) reported daily MRI patient contact. Slightly more than half (60.8%) had received formal MRI safety training, and 52.9% reported attending MRI safety courses during their academic studies (Table 1).

**TABLE 1:** Distribution of socio‐demographic and professional characteristics of nurses working in radiology departments (*n* = 153).

Characteristics	No. (%)
**Age (years)**	
20–29	36 (23.5)
30–39	88 (57.5)
40–49	20 (13.1)
50–59	9 (5.9)
**Sex**	
Male	51 (33.3)
Female	102 (66.7)
**Educational qualification**	
Diploma	26 (17.0)
Bachelor's	109 (71.2)
Master's	18 (11.8)
**Hospital sector**	
Public	123 (80.4)
Private	30 (19.6)
**Region of hospital**	
Central	91 (59.5)
Eastern	15 (9.8)
Northern	13 (8.5)
Southern	14 (9.2)
Western	20 (13.1)
**Years working in radiology**	
< 1	32 (20.9)
1–3	37 (24.2)
4–6	29 (19.0)
> 6	55 (35.9)
**Frequency of MRI patient contact**	
Daily	81 (52.9)
Weekly	36 (23.5)
Occasionally	36 (23.5)
**Received formal MRI safety training**	
Yes	93 (60.8)
No	60 (39.2)
**MRI safety courses during study**	
Yes	81 (52.9)
No	72 (47.1)

Internal consistency reliability was acceptable to good, with Cronbach's alpha coefficients of 0.716 for the knowledge domain and 0.837 for the attitude and practice domains.

### 3.2 Nurses' Knowledge of MRI Safety

Table 2 presents the distribution of nurses' responses to the MRI safety‐related knowledge items. Overall, nurses demonstrated a high level of knowledge across most MRI safety items. The majority correctly recognized that the MRI magnetic field is always on (87.6%), that ferromagnetic objects can become dangerous projectiles (87.6%) and that patients and staff must be screened for metallic objects before entering the MRI room (95.4%). High correct response rates were also observed for items related to MRI‐compatible equipment (90.2%), hearing protection (85.6%) and emergency procedures requiring patient removal from the MRI room (76.5%).

**TABLE 2:** Distribution of nurses' responses to MRI safety‐related knowledge items.

Items	Responses: no. (%)			*p* ^b^
	True	False	Don't know	
The clinical MRI magnetic field is always on, even when no scan is running.	110 (87.6)	24 (15.7)	19 (12.4)	< 0.0001
Ferromagnetic objects can become dangerous projectiles near the scanner.	134 (87.6)	9 (5.9)	10 (6.5)	< 0.0001
MRI uses ionizing radiation.^a^	34 (22.2)	84 (54.9)	35 (22.9)	< 0.0001
All patients and staff must be screened for metallic objects before entering the MRI room.	146 (95.4)	2 (1.3)	5 (3.3)	< 0.0001
Gradient magnetic fields can cause muscle twitching or nerve stimulation.	81 (52.9)	22 (14.4)	50 (32.7)	< 0.0001
Radiofrequency energy can cause burns if cables touch the skin or form loops.	90 (58.8)	20 (13.1)	43 (28.1)	< 0.0001
Hearing protection is required for all individuals who remain in the MRI room during scanning.	131 (85.6)	8 (5.2)	14 (9.2)	< 0.0001
Standard steel oxygen cylinders can safely be used inside the MRI scanner room.^a^	37 (24.2)	104 (68.0)	12 (7.8)	< 0.0001
Only MRI‐compatible medical equipment should be brought into the MRI scanner room.	138 (90.2)	6 (3.9)	9 (5.9)	< 0.0001
Pregnant staff are automatically prohibited from working anywhere in the MRI department.^a^	50 (32.7)	79 (51.6)	24 (15.7)	< 0.0001
In an emergency, resuscitation should be performed after moving the patient out of the MRI room.	117 (76.5)	15 (9.8)	21 (13.7)	< 0.0001
Medication patches with metallic backing should be removed before MRI.	121 (79.1)	3 (2.0)	29 (19.0)	< 0.0001
The MRI scanner is located in MRI safety zone number IV.	92 (60.1)	8 (5.2)	53 (34.6)	< 0.0001

^a^ False is the correct response in these cases.

^b^ Using chi‐square test for homogeneity.

However, knowledge gaps were identified in selected areas. For example, only 54.9% correctly identified that MRI does not use ionizing radiation, and 68% correctly recognized that standard steel oxygen cylinders are unsafe in MRI environments. Additionally, only 51.6% correctly responded that pregnant staff are not automatically prohibited from working in MRI departments.

For all knowledge items, the distribution of responses showed statistically significant differences (*p* < 0.0001), indicating that correct responses were significantly more frequent than incorrect or uncertain responses.

### 3.3 Nurses' Attitudes and Practices Related to MRI Safety

The majority of nurses demonstrated positive attitudes toward MRI safety. A large majority (79.1%) agreed or strongly agreed that their hospital provides clear MRI safety policies, that regular MRI safety training is essential (87.5%) and that they felt confident communicating with MRI technologists (83.0%). A high proportion (89.3%) also expressed willingness to attend annual MRI safety refresher sessions, and 82.3% agreed that incident reporting related to MRI is encouraged within their institutions.

Regarding practice, nurses reported strong adherence to MRI safety guidelines. Most participants agreed or strongly agreed that they assist patients in completing MRI safety checklists (92.1%), verify removal of metallic objects (93.5%), ensure MRI‐compatible equipment is used (92.1%) and provide appropriate hearing protection (85.7%). In addition, a large proportion indicated that they knew the emergency procedures (79.1%) and would report MRI‐related safety incidents or near misses (88.2%).

For all attitude and practice items, response distributions were statistically significant (*p* < 0.0001), indicating a strong overall tendency toward agreement and self‐reported compliance with recommended MRI safety practices (Table 3).

**TABLE 3:** Distribution of nurses' responses to attitude‐ and practice‐related items (nurses' responsibilities near MRI) in MRI.

Items	Responses: no. (%)					*p* ^a^
	Strongly disagree	Disagree	Neutral	Agree	Strongly agree	
**Attitudes**						
My hospital provides clear MRI safety policies.	—	6 (3.9)	26 (17.0)	89 (58.2)	32 (20.9)	< 0.0001
I feel confident communicating with MRI technologists about safety issues.	1 (0.7)	5 (3.3)	20 (13.1)	99 (64.7)	28 (18.3)	< 0.0001
Regular MRI safety training for nurses is essential.	—	5 (3.3)	14 (9.2)	79 (51.6)	55 (35.9)	< 0.0001
I can identify unsafe behaviour near the MRI scanner.	3 (2.0)	5 (3.3)	29 (19.0)	91 (59.5)	25 (16.3)	< 0.0001
I would attend annual MRI safety refresher sessions if available.	2 (1.3)	5 (3.3)	9 (5.9)	89 (58.2)	48 (31.1)	< 0.0001
Incident reporting related to MRI is encouraged in my hospital.	4 (2.6)	5 (3.3)	18 (11.8)	90 (58.8)	36 (23.5)	< 0.0001
**Practice (nurses' responsibilities near MRI)**						
I assist patients in completing the MRI safety checklist before the scan.	1 (0.7)	4 (2.6)	7 (4.6)	105 (68.6)	36 (23.5)	< 0.0001
I verify that all jewellery and metallic items are removed from patients before entering the MRI scanner.	1 (0.7)	2 (1.3)	7 (4.6)	100 (65.4)	43 (28.1)	< 0.0001
I ensure IV lines, monitors or devices I use are MRI‐compatible or remain outside the scanner room.	—	—	12 (7.8)	94 (61.4)	47 (30.7)	< 0.0001
I make sure patients receive and correctly wear hearing protection.	—	6 (3.9)	16 (10.5)	98 (64.1)	33 (21.6)	< 0.0001
I know the emergency steps to follow if a patient deteriorates during MRI.	—	8 (5.2)	24 (15.7)	95 (62.1)	26 (17.0)	< 0.0001
I report any MRI‐related safety incident or near miss I observe.	1 (0.7)	1 (0.7)	16 (10.5)	103 (67.3)	32 (20.9)	< 0.0001

^a^ Using chi‐square test for homogeneity.

### 3.4 Comparison of Mean Knowledge, Attitude and Practice Scores

Table 4 presents comparisons of mean KAP scores according to nurses' socio‐demographic and professional characteristics. Mean knowledge scores differed significantly by age group (*p* = 0.017), with higher scores observed among nurses aged 40–49 years and 50–59 years than in younger age groups. The effect size (*η* ^2^ = 0.066) indicates a medium magnitude of difference across the four age groups.

**TABLE 4:** Comparison of mean values of nurses' knowledge, attitude and practices scores in relation to their socio‐demographic and professional characteristics.

Characteristics	Knowledge scores			Attitude scores			Practice scores		
	Mean (SD)	*t*/*F*‐value	*p*	Mean (SD)	*t*/*F*‐value	*p*	Mean (SD)	*t*/*F*‐value	*p*
**Age (years)**									
20–29	8.42 (2.9)	3.522	0.017 (0.066)^a^	23.47 (1.9)	2.329	0.077	23.25 (2.9)	5.828	0.001 (0.105)^a^
30–39	9.26 (2.9)			24.02 (3.2)			24.76 (2.7)		
40–49	10.75 (1.7)			25.55 (2.8)			26.30 (3.3)		
50–59	10.44 (1.9)			24.22 (2.2)			23.44 (2.1)		
**Sex**									
Male	8.78 (2.7)	1.673	0.096	23.96 (3.1)	0.434	0.665	24.16 (3.2)	1.113	0.267
Female	9.59 (2.9)			24.18 (2.8)			24.72 (2.8)		
**Educational qualification**									
Diploma	9.35 (3.4)	0.650	0.523	23.65 (2.5)	0.660	0.518	24.74 (2.2)	1.687	0.189
Bachelor's	9.44 (2.7)			24.28 (2.8)			24.42 (4.2)		
Master's	8.61 (3.1)			23.72 (3.7)			23.39 (4.1)		
**Hospital sector**									
Public	9.56 (2.9)	−2.078	0.039 (0.423)^a^	23.93 (2.9)	1.547	0.124	24.72 (2.9)	−1.599	0.112
Private	8.37 (2.5)			24.83 (2.8)			23.77 (3.1)		
**Region of hospital**									
Central	9.59 (2.7)	1.768	0.138	24.03 (2.8)	1.513	0.201	24.54 (2.7)	0.153	0.962
Eastern	10.13 (3.8)			24.07 (2.1)			24.07 (4.7)		
Northern	7.76 (2.9)			23.08 (3.4)			24.46 (1.8)		
Southern	8.64 (3.1)			23.71 (3.2)			24.57 (3.1)		
Western	9.0 (2.4)			25.40 (3.1)			24.85 (3.1)		
**Years working in radiology**									
< 1	8.68 (3.0)	1.476	0.223	23.25 (2.4)	1.439	0.234	24.03 (2.4)	1.177	0.321
1–3	8.92 (3.4)			24.14 (2.6)			24.05 (3.2)		
4–6	9.93 (2.0)			24.72 (3.0)			25.03 (2.3)		
> 6	9.65 (2.7)			24.25 (3.2)			24.87 (3.3)		
**Frequency of MRI patient contact**									
Daily	10.22 (2.1)	9.414	< 0.001 (0.112)^a^	24.23 (2.7)	0.411	0.664	24.80 (3.1)	0.749	0.475
Weekly	8.33 (3.3)			24.19 (2.6)			24.19 (2.9)		
Occasionally	8.30 (3.2)			23.72 (3.5)			24.25 (2.6)		
**Received formal MRI safety training**									
Yes	10.06 (2.6)	−4.193	< 0.001 (0.694)^a^	24.49 (2.5)	−2.101	0.037 (0.348)^a^	24.94 (2.7)	−2.161	0.032 (0.358)^a^
No	8.18 (2.8)			23.50 (3.3)			23.90 (4.1)		
**MRI safety courses during study**									
Yes	9.58 (2.6)	−1.167	0.245	24.49 (2.7)	−1.779	0.077	24.49 (2.4)	0.159	0.874
No	9.04 (3.1)			23.67 (3.1)			24.57 (3.4)		

^a^ Effect size.

The mean knowledge scores of nurses were significantly higher among those working in the public sector than in those in the private sector (*p* = 0.039), with a medium effect size of 0.423. Knowledge scores were also significantly higher among nurses who had daily contact with MRI patients (*p* < 0.001) and those who had received formal MRI safety training (*p* < 0.001), with effect sizes of 0.112 and 0.694, respectively.

No statistically significant differences in knowledge scores were found by sex, educational qualification, region, years of radiology experience or undertaking MRI safety courses during their study.

Attitude scores showed no statistically significant differences across most socio‐demographic and professional variables. However, nurses who had received formal MRI safety training demonstrated significantly higher attitude scores (*p* = 0.037) than those who had not, with a medium effect size of 0.348.

Practice scores varied significantly by age group (*p* = 0.001), with a medium effect size of 0.105, where higher scores were reported among nurses aged 40–49 years. Additionally, nurses who had received formal MRI safety training had significantly higher practice scores (*p* = 0.032), with an effect size of 0.358. No significant differences in practice scores were observed based on sex, educational qualification, hospital sector, region, years of experience, frequency of MRI patient contact or MRI safety courses during their study.

### 3.5 Multiple Linear Regression Analysis

Multiple linear regression analysis was conducted to identify factors independently associated with MRI knowledge scores. The overall model was statistically significant, *F*(7, 145) = 5.705, *p* < 0.001, and explained 21.6% of the variance in knowledge scores (*R* ^2^ = 0.216; adjusted *R* ^2^ = 0.178).

After adjustment for the variables included in the model, age 40–49 years, daily MRI patient contact and formal MRI safety training were significant independent predictors of knowledge scores. Nurses aged 40–49 years had significantly higher knowledge scores than those aged 20–29 years (*B* = 1.675, *β* = 0.198, *t* = 2.276, *p* = 0.024; 95% CI: 0.220–3.129). Nurses with daily MRI patient contact had significantly higher knowledge scores than those with occasional contact (*B* = 1.351, *β* = 0.237, *t* = 2.532, *p* = 0.012; 95% CI: 0.297–2.406). In addition, nurses who had received formal MRI safety training had significantly higher knowledge scores than those who had not received training (*B* = 1.331, *β* = 0.228, *t* = 2.957, *p* = 0.004; 95% CI: 0.441–2.220). The results of the multiple linear regression analysis are presented in Table 5.

**TABLE 5:** Multiple linear regression analysis of factors associated with MRI knowledge scores.

Variable	*B*	SE	*β*	*t*‐value	*p*	95% CI
Age 30–39 years	0.714	0.514	0.124	1.389	0.167	−0.302 to 1.730
Age 40–49 years	1.675	0.736	0.198	2.276	0.024	0.220 to 3.129
Age 50–59 years	1.678	0.970	0.139	1.730	0.086	−0.239 to 3.595
Hospital sector (private vs. public)	0.661	0.541	0.092	1.222	0.224	−0.408 to 1.730
Daily MRI patient contact	1.351	0.534	0.237	2.532	0.012	0.297 to 2.406
Weekly MRI patient contact	0.041	0.614	0.006	0.067	0.947	−1.172 to 1.254
Received formal MRI safety training	1.331	0.450	0.228	2.957	0.004	0.441 to 2.220

*Note:* Model statistics: *R* ^2^ = 0.216; adjusted *R* ^2^ = 0.178; *F*(7, 145) = 5.705; *p* < 0.001.

Abbreviations: β, standardized regression coefficient; *B*, unstandardized regression coefficient; CI, confidence interval; SE, standard error.

## 4 Discussion

This multicentre national study assessed MRI safety‐related KAP among nurses in Saudi radiology departments. The findings indicate strong procedural adherence and positive safety attitudes but variable conceptual knowledge.

High levels of knowledge were observed in domains directly linked to daily clinical workflow, including equipment compatibility, metallic object screening, projectile hazards and emergency preparedness. These areas constitute the most visible and protocol‐driven components of MRI risk mitigation and are consistently emphasized in international safety guidelines (Expert Panel on MR Safety et al. 2013; Pedrosa et al. 2025). The strong performance in these domains suggests that procedural standardization and control of access to MRI environments through established zoning practices within Saudi radiology departments are functioning effectively. Repeated exposure to screening protocols and zoning practices likely reinforces the retention of safety‐related behaviours through experiential learning.

In contrast, conceptual gaps were evident in terms of the fundamental physics behind MRI. Only slightly more than half of the participants correctly identified that MRI does not use ionizing radiation, and knowledge regarding pregnancy‐related work policies and the use of standard steel oxygen cylinders was inconsistent. Similar misconceptions have been documented internationally. For example, Abd Aziz et al. (2022) reported confusion between MRI and ionizing radiation modalities among healthcare professionals in Malaysia, while Al‐Radaideh and Al‐Modallal (2023) observed variability in theoretical knowledge about MRI safety in Jordan, despite positive attitudes regarding safety. Moreover, within Saudi Arabia, Alghamdi et al. (2021) identified moderate knowledge levels among nurses in two government hospitals, particularly in terms of physics‐related concepts. The present multicentre study extends these findings nationally and confirms that the reinforcement of fundamental concepts remains a priority. Misattribution of the risk of ionizing radiation to MRI is clinically significant, as it may influence patient counselling and risk perception. National patient‐level research has also demonstrated limited awareness of MRI safety principles prior to scanning (Alahmari et al. 2022), suggesting that provider uncertainty may contribute to persistent misconceptions among the general public.

Attitudinal alignment with MRI safety principles was strong across the 15 institutions included in the current study. Most nurses agreed that clear safety policies are in place, that regular training is essential and that incident reporting is encouraged. These findings indicate that safety culture is broadly embedded within Saudi Arabian radiology practice settings. International research similarly demonstrates high perceived preparedness among MRI professionals, even when objective knowledge varies (Mittendorff et al. 2023). This reinforces the theoretical distinction between safety culture and safety competence, suggesting that shared safety values and behavioural commitment do not necessarily equate to a comprehensive understanding of the fundamental physics behind MRI technology.

Self‐reported practice adherence was consistently high across the domains of screening, verification of equipment compatibility, hearing protection enforcement, emergency preparedness and incident reporting. Practice scores were significantly higher among nurses aged 40–49 years and those who had received formal MRI safety training. The alignment between positive attitudes and strong reported practices supports established safety science models in which institutional culture reinforces behavioural compliance. However, because practice measures were self‐reported, the potential for social desirability bias must be acknowledged. Safety research consistently demonstrates discrepancies between reported and observed compliance in high‐risk healthcare environments (Reason 2000). Future studies incorporating observational audits or simulation‐based evaluation would provide a more objective assessment of operational performance.

In bivariate analyses, knowledge was associated with age group, hospital sector, frequency of MRI patient contact and receipt of formal MRI safety training. After multivariable adjustment, age 40–49 years, daily MRI patient contact and formal MRI safety training remained significant independent predictors of knowledge scores. The association observed among nurses aged 40–49 years may reflect the cumulative effect of professional experience, repeated exposure to MRI‐related workflows and greater familiarity with institutional safety procedures acquired over time. These findings suggest that MRI safety knowledge is influenced by both experiential exposure and structured education. Daily interaction with MRI patients may reinforce knowledge through repeated engagement with screening procedures and safety protocols, while formal MRI safety training may strengthen awareness of MRI‐specific hazards beyond routine clinical exposure. These findings are consistent with previous studies reporting higher levels of MRI safety preparedness and confidence among healthcare professionals who receive structured MRI safety education (Mittendorff et al. 2023). Furthermore, repeated engagement in complex technological environments has been shown to enhance hazard recognition and situational awareness (Flin and O'Connor 2017), whereas unfamiliarity with modality‐specific hazards has been identified as a contributor to preventable MRI‐related incidents (Expert Panel on MR Safety et al. 2013; Shah and Aran 2023).

The present findings have important implications for nursing practice and MRI safety education by underscoring the importance of strengthening foundational MRI safety knowledge alongside maintaining positive safety attitudes and practices. The observed knowledge gaps, particularly in physics‐related concepts, support the need for structured educational initiatives and regular MRI safety training programmes for nurses working in radiology departments. At the institutional level, integrating MRI safety standards into continuing professional development and nursing governance frameworks may further enhance patient and staff safety within MRI environments.

From the perspectives of radiology nursing competency, institutional safety training and quality improvement, periodic assessment of MRI safety‐related KAP may serve as a valuable tool for monitoring staff competency and identifying areas requiring additional educational support. Integrating KAP assessments into routine MRI safety training programmes and institutional quality assurance activities may help evaluate the effectiveness of educational interventions and support continuous improvement in MRI safety performance.

Limitations of this study warrant consideration. Although the cross‐sectional design was appropriate for assessing MRI safety‐related knowledge, attitudes, and practices and identifying independent predictors of MRI safety knowledge, causal relationships between professional characteristics and knowledge outcomes cannot be established, and temporal associations between exposure variables and competency development cannot be determined. Data were derived from self‐administered questionnaires, which are subject to recall bias and social desirability bias, particularly in the assessment of reported safety practices. In addition, the assessment relied on self‐reported knowledge, attitudes and practices rather than objective competency‐based evaluation, which may not fully reflect actual MRI safety performance in clinical practice. Consequently, actual behavioural adherence in clinical settings may differ from reported responses. Although the study was multicentre in nature and the included institutions were distributed across the country, participation was voluntary, which may have introduced selection bias by favouring individuals with greater interest in or awareness of MRI safety. Furthermore, the effects on MRI safety competence of institutional determinants, such as the presence of designated MRSOs, formalized safety governance structures, zoning audit frequency and structured refresher training programmes, were not directly assessed. The absence of these variables limits the ability to fully evaluate organizational influences on MRI safety competence. In addition, the use of an adapted questionnaire may limit direct comparability with findings from studies that used different MRI safety assessment instruments.

## 5 Conclusion

This national multicentre study demonstrates that MRI safety culture among nurses in Saudi radiology departments is well established at the procedural level, with high adherence to operational safety practices. However, gaps in conceptual knowledge persist, particularly regarding the non‐ionizing nature of MRI and pregnancy‐related policies. Older age, daily MRI patient contact and formal MRI safety training were significant predictors of knowledge, highlighting the complementary roles of experiential exposure and structured education in strengthening MRI safety competence. Strengthening structured, competency‐based education through periodic reassessment may further enhance conceptual understanding and long‐term safety performance among nurses working in MRI environments.

## Author Contributions

Mohammed A. Alolayani and Sami A. Alghamdi conceived and designed the study. Mohammed A. Alolayani collected the data with support from Sami A. Alghamdi. Mohammed A. Alolayani and Sami A. Alghamdi contributed to data management and interpretation of the findings. Mohammed A. Alolayani prepared the initial draft of the manuscript. Sami A. Alghamdi supervised the study, contributed to data analysis, critically revised the manuscript and secured the research funding. Othman I. Alomair, Dhafer M. Alahmari and Wejdan M. Arif contributed to the review and revision of the manuscript. All authors reviewed and approved the final version of the manuscript.

## Funding

This research was funded by the Deanship of Scientific Research at King Saud University through the Ongoing Research Funding Program, grant number ORF‐2026‐2130, Riyadh, Saudi Arabia.

## Disclosure

No AI tools were used in the preparation of this manuscript.

## Ethics Statement

Ethical approval for this study was obtained from the Institutional Review Board of King Saud University Medical City (Project No. E‐25‐10522). The study adhered to the principles of the Declaration of Helsinki.

## Consent

Participation was voluntary, and electronic informed consent was obtained from all participants prior to data collection.

## Conflicts of Interest

The authors declare no conflicts of interest.

## Data Availability

The data that support the findings of this study are available on request from the corresponding author. The data are not publicly available due to privacy or ethical restrictions.
